# Appropriate scenarios for mercury emission control from coal-fired power plant in Thailand: emissions and ambient concentrations analysis

**DOI:** 10.1016/j.heliyon.2020.e04197

**Published:** 2020-06-12

**Authors:** S. Thepanondh, V. Tunlathorntham

**Affiliations:** a Department of Sanitary Engineering, Faculty of Public Health, Mahidol University, Bangkok, 10400, Thailand; b Center of Excellence on Environmental Health and Toxicology (EHT), Bangkok, 10400, Thailand

**Keywords:** Coal combustion, Co-benefit, iPOG, Mercury emission, Atmosphere modelling, Air quality, Environmental assessment, Environmental impact assessment, Sustainable development, Environmental science

## Abstract

Optimum control of mercury released from the coal-fired power plant is evaluated by determining its efficiency and appropriateness in reducing emissions and ambient air concentrations. The 2400 MW power plant fueled by lignite located in Thailand is demonstrated in this study. Emissions of mercury from the coal-fired power plant are calculated under 3 major scenarios. The first scenario is the amount of mercury released under the existing operation of the power plant. Emission rate of mercury is calculated as 41 g/h which indicates a co-benefit of mercury removal from the installation of existing conventional air pollution treatment systems (electrostatic precipitator and wet flue gas desulfurization) as compare with the 2^nd^ scenario of without equipping of air pollution control devices at the power plant (374 g/h of mercury emission). Adding controlling measures to existing operation of the power plant can lead to decreasing of mercury emissions at different levels. The relationship between changing of emissions affected to ambient air concentrations of mercury is evaluated using the CALPUFF air dispersion model. Results indicate small decreasing of predicted ambient concentrations after applying additional mercury control measures to the BAU of the power plant. This study reveals the co-benefit of existing air pollution treatment devices in controlling mercury emission. It also illustrates that the efficiency and appropriateness of current air pollution control system is in an optimal and acceptable levels in mercury control.

Finding and methodology in this study can be used as a case study in quantitative evaluation of the effectiveness and appropriateness of environmental control mitigation measures added to the existing operations. It clearly illustrates the need to analyze the co-benefit of current air pollution control system towards the accomplishment on controlling emissions of other emerging air pollutants which will provide the best optimum air pollution control to the emission source.

## 1 Introduction

Mercury and mercury compounds are toxic to humans and the environment. Gaseous mercury is considered as global pollutant since it has a long atmospheric lifetime and persistence in the environment. Mercury is emitted from both the natural sources, weathering of rocks, forest fires, soil erosion and the anthropogenic sources (United Nations Environment Programme, 2008).

The major source of anthropogenic mercury emissions in atmosphere is the coal combustion which accounting about 45% of the total mercury emission (Dziok et al.2015; United Nations Environment Programme, 2008). Mercury released from this source can be appered in 3 phases. They are 1) particle-bound mercury (Hg_p_), 2) vapor-phase oxidized mercury (Hg^2+^) and 3) vapor-phase elemental mercury (Hg^0^) which is normally can be converted into Hg^2+^ or Hg_p_ (Lopez-Anton et al., 2010; US Environmental Protection Agency, 2002).

Controlling of mercury from coal combustion can be managed by both pre and post combustion management (Dziok et al., 2015). The pre-combustion controls include coal washing and fuel switching which aims to reduce the amount of mercury in the fuel (Hu and Cheng, 2016). Managing of combustion process and installation of air pollution control equipment are used as the post-combustion measures to control mercury emission (Wang et al., 2014).

Controlling of the mercury emitted from coal-fired power plants are considered to be as co-benefit removal from the installation of pollution treatment systems aimed in controlling the conventional air pollutants. Oxidize mercury is water-soluble and can be removed in wet scrubber such as wet flue gas desulfurization systems (WFGD). A particle-bound mercury (Hg_p_) is almost completely capture in particulate control equipment such as electrostatic precipitator (ESPs) and fabric filters (FFs) while elemental mercury is not usually trapped by emission control equipment and is emitted into the atmosphere. Therefore, controlling of mercury emission can be successfully achieved by selecting of the suitable and appropriate technology to reduce its emission (Burmistrz et al., 2016; Naik et al., 2009; U.S. Environmental Protection Agency, 2002).

The main objective of this research is to evaluate the appropriate and optimum methodology in controlling of mercury emission from the coal-fired power plant which will be resulted in managing of ambient mercury concentrations. Emissions of mercury are estimated using the iPOG model under various control measures and be used as input data to calculated the ambient concentrations using the CALPUFF air dispersion model.

## 2 Methodology

### 2.1 Site description

The Mae Moh coal-fired power plant selected in this analysis is located about 616 km in the northern direction from Bangkok, Thailand. The first Mae Moh lignite-fired power plant with 75 MW began construction in 1975 and was completed in 1978. The next 2 × 75 MW units started commercial operation in 1979 and 1981, respectively. The 4 × 150 MW plant started commercial operation from 1984 to 1985. Power plant units 8–13 (with 300 MW each) were constructed and commissioned from 1989 to 1995. In 1999, the facility's unit 3 was decommissioned, while units 1 and 2 were retired in 2000. Today, the plant has a total of 2,400 MW generating capacity in operation (ERIA, 2017). Currently, they are 10 active electricity generating units. Seven of them have the electricity generating capacity of 150 MW each while other 3 units have the capacity of 300 MW each This production capacity can supply 50% of the electricity to the northern area, 30% to the central area, and 20% to the northeastern area of Thailand. Lignite is fueled to generate 2400 MW electricity. The fuel consumption is approximately 16 million tons yearly. Lignite is mined from the adjacent Mae Moh coal field, and the character of this domestic lignite is low calorie and high sulfur content. Level of sulfur content of this lignite are ranging from 2.2 to 3.1 % with an average of about 2.5 %, 17–27 % ash, and 2,639 kcal/kg, respectively (Punyawadee et al., 2006). However, high variations of the level of sulfur content of lignite combusted this power plant were reported by Watchalayann et al. (2018). Measured sulfur contents carried out under this study were in the range of 6.28–8.12% as presented in Table 1. In this study, samplings of coal were conducted based on the ASTM D 4596-09 procedure (Standard practice for collection of channel samples of coal in a mine). Collected samples were then prepared for analysis following the ASTM D2013-03 method and sulfur content was analyzed following the ASTM D4239-14. Consequently, when the lignite is combusted, emission of SO_2_ is high, with an estimated annual emission of approximately 540,000 tons per year. The power plant is located in the valley with the complex terrain characteristic. High hills surround the Mae Moh valley, particularly to the East and West. Passing almost North-South along the East is a huge limestone ridge. Therefore, peak concentrations of air pollutants (SO_2_) measured at the ground level in some villages located in the vicinity of the power plant were occasionally observed during the incidence of surface temperature inversion.

**Table 1:** Proximate and chemical analysis of feed coal (lignite) (Watchalayann et al., 2018).

Property	Value	Unit
Inherent moisture	15.83–19.06	% content
Total moisture	30.57–32.49	
Volatile matter	42.58–43.06	
Ash	31.45–32.18	
Fixed carbon	25.24–25.49	
Carbon (C)	47.55–48.86	
Hydrogen (H)	5.67–6.28	
Nitrogen (N)	1.77–1.78	
Sulfur (S)	4.46–4.70	
Oxygen (O)	7.17–8.12	
Chlorine (Cl)	0.09–0.15	g/kg
Barium (Ba)	0.10–0.26	
Sodium (Na)	9.21–12.49	
Calcium (Ca)	93.36–116.44	
Bromine (Br)	<1.00	mg/kg
Arsenic (As)	290.12–411.63	
Selenium (Se)	0.76–1.29	
Mercury (Hg)	132.31–198.78	μg/kg

High concentrations of heavy metals were also reported from both fly and bottom ash after the flue gas be treated by the air pollution control devices (electrostatic precipitator for particulate control and wet flue gas desulfurization for sulfur dioxide control). Arsenic, Co, Cr, Ni, Mo and Sb generally increase in concentration going from bottom ash through the sequence of electrostatic precipitator ashes and reach maxima of As (352 ppm), Co (45 ppm), Cr (105) ppm, Mo (32 ppm), Ni (106 ppm) and Sb (15 ppm) in the electrostatic precipitator ashes (Hart et al., 1995). Proximate analysis and chemical property of lignite used in this power plant is presented in Table 1.

Mercury content in the lignite was analyzed following the ASTM D6722-11 method. The US.EPA method 29 was employed to determine mercury emission from the combustion stacks of the power plant. It was reported that concentrations of mercury emitted from Mae Moh power plant were in the range of 5.22–9.20 μg/m^3^. Mostly of them (about 70%) were presence in the form of elemental mercury (Hg^0^). The proportion of oxidized mercury (Hg^2+^) was about 20–25% while the particulate mercury (Hg_p_) was about 5% from total mercury concentrations, respectively (Watchalayann et al., 2018). This measured data will be further used to discuss with the results obtained from the analysis under this study (section 3.1).

### 2.2 Emission and air dispersion model

The iPOG model (The Interactive Process Optimization Guidance) developed by Niksa Energy Associates LLC for the United Nations Environment Program is applied to evaluate the mercury emission rate and type of mercury from the full-scale gas cleaning system in this study. iPOG is a software suite that uses the “tree decision” concept in a POG document designed as a model for which users can change the parameters (United Nations Environmental Programme, 2012). Emission rate of mercury after applying various control measures can be estimated by the model. An example of those measures are such as coal blend, coal properties, coal conditioning, selection of gas cleaning and conditioning systems including the use of common technologies of other gas cleaning systems such as existing pollution control devices for NOx, PM and SO_2_ or mercury-specific control technologies such as halogenation agent, injection carbon sorbents to find effective mercury control and reduce the emission rate of mercury into the atmosphere.

**Figure 1:**
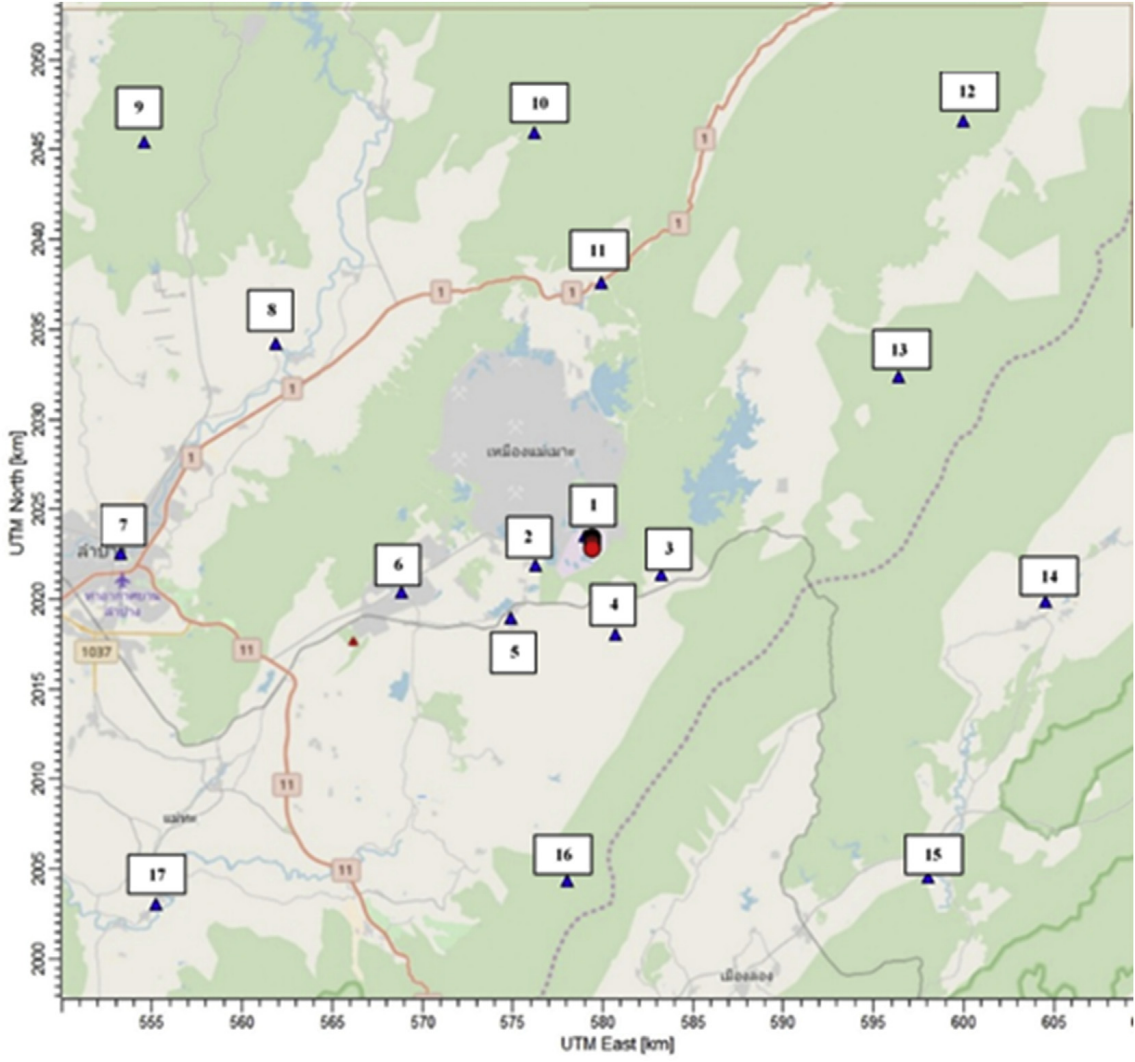
Locations of receptor and source of coal-fired power plant.

Emission rates under each control scenarios are used as an input data to predict the ambient mercury concentration by the CALPUFF dispersion model. It is a non-steady state puff model that can be computed in hours-by-hour under the spatial variations of the wind. This model has been evaluated for its accuracy in predicting the distribution of air pollutants from few kilometers to several kilometers (Scire et al., 2000). Prognostic meteorological data used in this study is simulated by the WRF model (Weather Research Forecast model) coupled with on-site measured meteorological characteristics for the year 2019. The US.EPA approved version of CALPUFF (version 5.8.5) is used for the simulation in this analysis. The study domain is designed for grid center coordinate at Latitude 18′29″N and Longitude 99′75″E with a grid spacing of 1 × 1 km^2^, cover an area of 30 × 30 km^2^. Mercury emissions are calculated from 10 point sources in the unit of gram per hour (g/hr) and the ground level ambient concentrations are predicted in the unit of nanogram per cubic meter (ng/m^3^) as an average of 1 year. Ambient ground level concentrations of mercury at 17 discrete receptors located in the surrounding areas in the distance of 0.7–33.5 km from the power plant are predicted by the CALPUFF model as illustrated in Figure 1.

## 3 Results and discussion

### 3.1 Mercury emission analysis

Emissions of mercury from the coal-fired power plant are calculated under 3 major scenarios. The first scenario is the amount of mercury released under the existing operation of the power plant. Emission rate of mercury is calculated as 41 g/h which indicates a co-benefit of mercury removal from the installation of existing conventional air pollution treatment systems (electrostatic precipitator: ESPc and wet flue gas desulfurization; WFGD) as compare with the 2^nd^ scenario of without equipping of air pollution control devices at the power plant (374 g/h of mercury emission under the 2^nd^ scenario). Emissions of mercury from various mercury control measures added from the existing operation (the 1^st^ scenario) are calculated under the 3^rd^ scenario. Results from the iPOG model indicate that the best technology in controlling of mercury released from this power plant can be achieved by adding of the brominated activated carbon injection system to its existing operation. Emission of mercury under this measure is calculated as 10.44 g/h (about 4 times decreasing from the existing release). Increasing of 87.2% control efficiency is due to fact that the activated carbon injection (ACl) is an effective adsorbent due to its small porosity and high surface area, which can adsorb pollutants and chemical reactions (Ancora et al., 2015). Details of speciated mercury emission estimated under each scenario is also presented in Table 2.

**Table 2:** Speciated mercury emission.

Scenario	Stack mercury emission		Stack mercury speciation (%)		Overall Hg removal efficiency (%)
	(g/h)	g/TJ	Hg^2+^	Hg^0^	
1	41	0.7	39	60	89
2	374	6.4	17	80	0.5
3	10.44	0.1	38	60	97

These results are coincided with measured data by Watchalayann et al. (2018) previously presented in section 2.1. Mostly of mercury released from the stack emission are presence in the form of elemental mercury (Hg^0^). The existing air pollution control devices of the power plant can remove mercury from flue gases in two ways: removal of Hg_p_ in particulate control devices and removal of Hg^2+^ in wet FGD scrubbers. Thus, the mercury removed from the flue gas may be found in fly ash and in the scrubber solids (gypsum of the wet flue gas desulfurization). This mercury waste is considered as the hazardous waste and can be further manage through the industrial waste management procedure. High sulfur content of lignite also enhance and inhibit the removal process of each speciated mercury during the various stage of emission control devices. The Hg^0^ oxidation in the flue gas would be conducive while the adsorption of this elemental mercury on the surface of the fly ask would be inhibited. However, it will enhance the process of Hg^2+^ removal in the wet flue gas desulfurization system.

**Figure 2:**
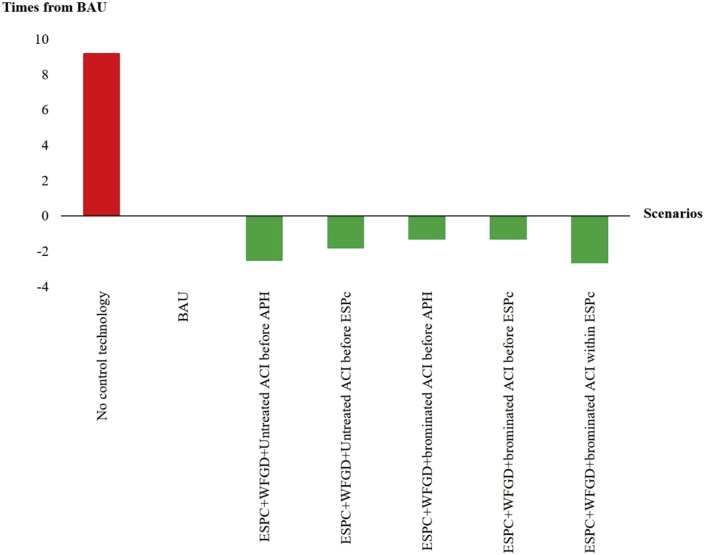
Changing of mercury emission after adding of pollution control devices to existing system.

We further analyze amount of mercury emission from several mercury management strategies by adding a technology in controlling mercury to the existing air pollution control devices. Results are presented as the number of decreasing/increasing times over the existing emission amount as illustrated in Figure 2.

There are several air pollution control devices and procedures (such as coal washing and chemical cleaning) used in controlling the release of industrial air pollutants. Some technologies are specifically invented for mercury treatment. Analytical results reveal that mostly of the technologies related to coal cleaning and halogen injection can provide positive results in decreasing of mercury emission from its current level as illustrated by the green colored bar in Figure 2. Mercury reduction due to coal cleaning is an effective alternative in reducing the release of mercury into the atmosphere (Hu and Cheng, 2016; Huang et al., 2017) considered as pre-combustion control which able to reduce the average concentration of mercury in unprocessed coal by 30% (Tang et al., 2007; Streets et al., 2005; Ancora et al., 2016). It will also benefit in decreasing the mercury content in combustion ash plus increasing heating values and the efficiency of the coal combustion process. It should be noted that coal washing is also the least costly method for pre-combustion control (Hu and Cheng, 2016).

As for halogen injections, such as adding chlorine or bromine, is based on the knowledge that the air pollution control devices will have higher performance in treating of mercury when the amount of chlorine in the coal is increased (Wang et al., 2009). The study carried out by Ancora et al. (2015) reported that the performance of air pollution control devices could be increased from 92% - 97% by adding 25 ppm of CaBr_2_ in coal combustion. Chlorine is a strong oxidizing agent to convert elemental mercury to oxidized mercury (Liu et al., 2007; Qu et al., 2009) while bromine is shown to be more effective in the oxidation of mercury in the flue gas than chlorine (Liu et al., 2007; Van Otten et al., 2011). This is due to the fact that chlorine is present mostly as HCl with a small fraction as the reactive Cl, while Br and HBr fraction are comparable at the flame temperatures where homogeneous of elemental mercury occur (Niksa et al., 2010). However, the addition of chlorine and bromine in coal has shown to be effective in mercury removal for coal-fired power plants equipped with dust control equipment with only 6% increase in cost (United Nations Environmental Programme, 2010; Ancora et al., 2015).

Selective catalytic reduction (SCR) is the technology used in controlling of oxides of nitrogen emission. However, this technology is associated with the speciation of mercury in flue gas, as the SCR can oxidize Hg^0^ to Hg^2+^ and then be captured by WFGD system (Ancora et al., 2015). Increasing of mercury removal is due to the fact that Hg^0^ has chemical inertness, high volatility and poor water solubility (United Nations Environmental Programme, 2010). A study by the China Council for International Cooperation on Environment and Development (CCICED, 2011) reported that SCR + ESPc + WFGD has a mercury removal efficiency of 66%, The control efficiency is slightly higher than the ESPc + WFGD system. Previous study by Zhao et al. (2017) also report that mercury removal efficiency of SCR + ESPc + WFGD was very close to ordinally ESPc + WFGD.

In this study. It is found that adding the SCR to existing air pollution control devices is not affected in better performance in mercury removal. This can be explained by high chlorine content (0.09–0.15 g/kg) of the lignite that enhances the major removal activity to be achieved from the halogenic reaction.

### 3.2 Spatial distribution of mercury concentrations

**Figure 3:**
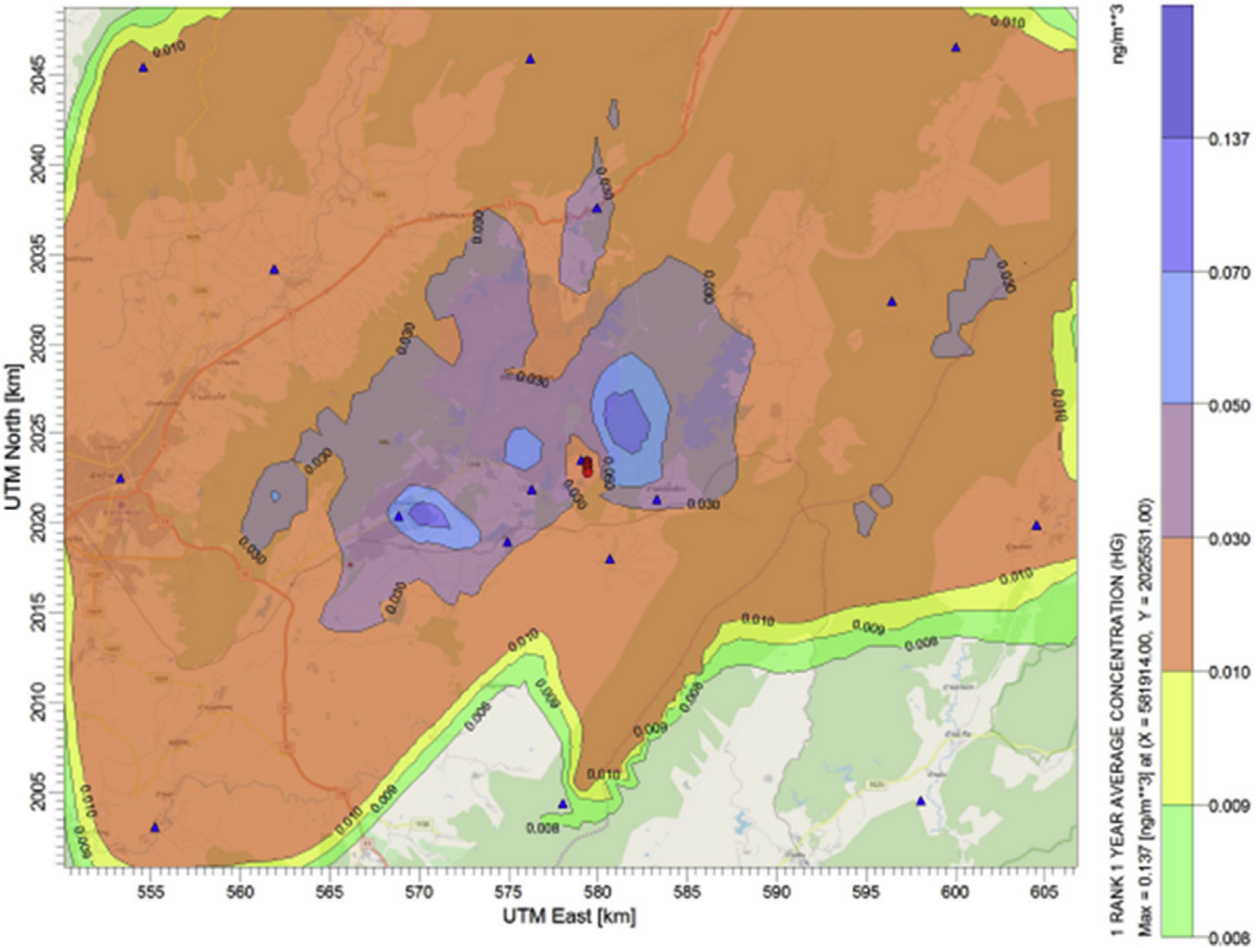
Annual average mercury concentration (ng/m^3^) under “BAU scenario”.

**Figure 4:**
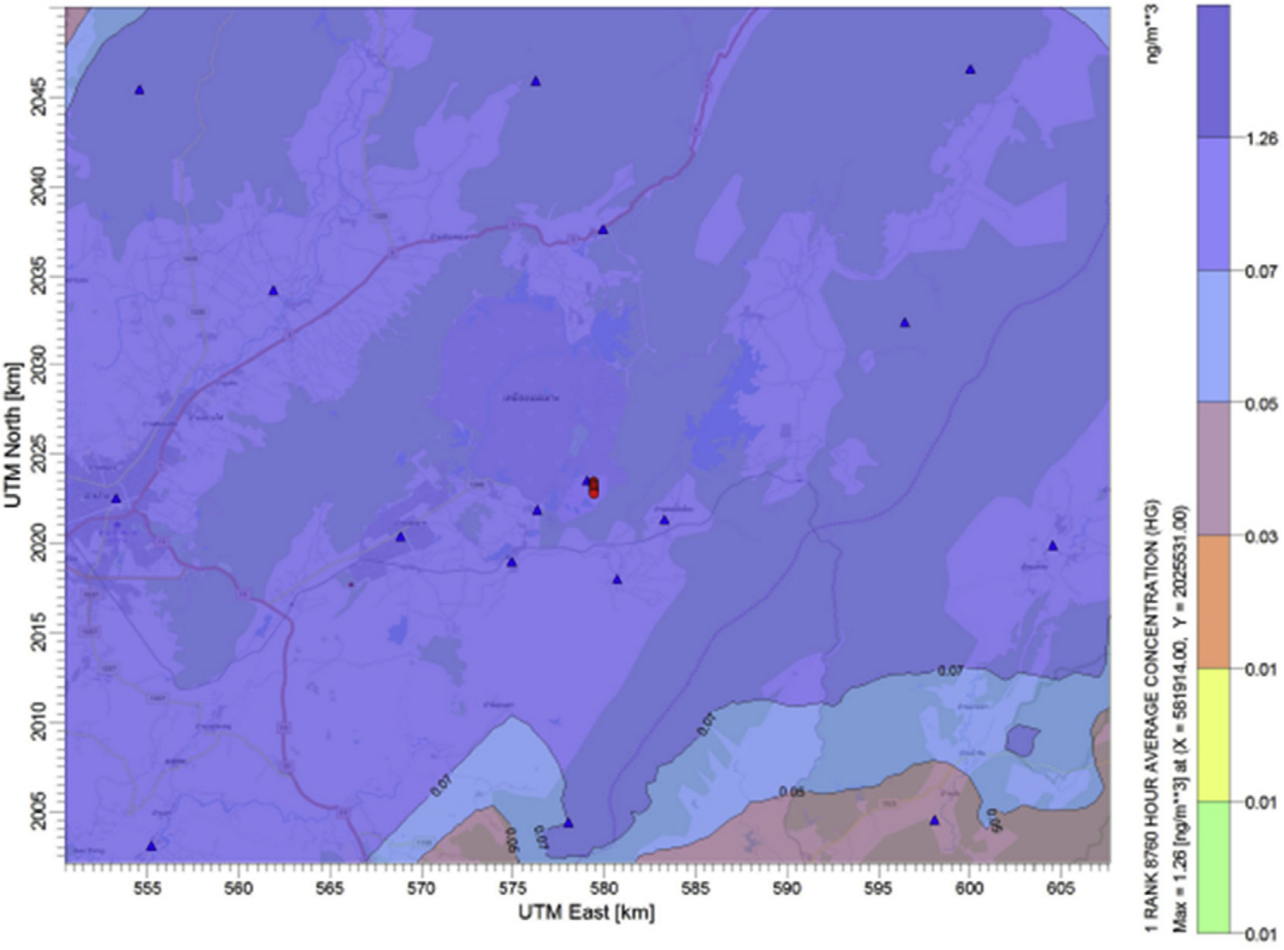
Annual average mercury concentration (ng/m^3^) under “no control technology”.

**Figure 5:**
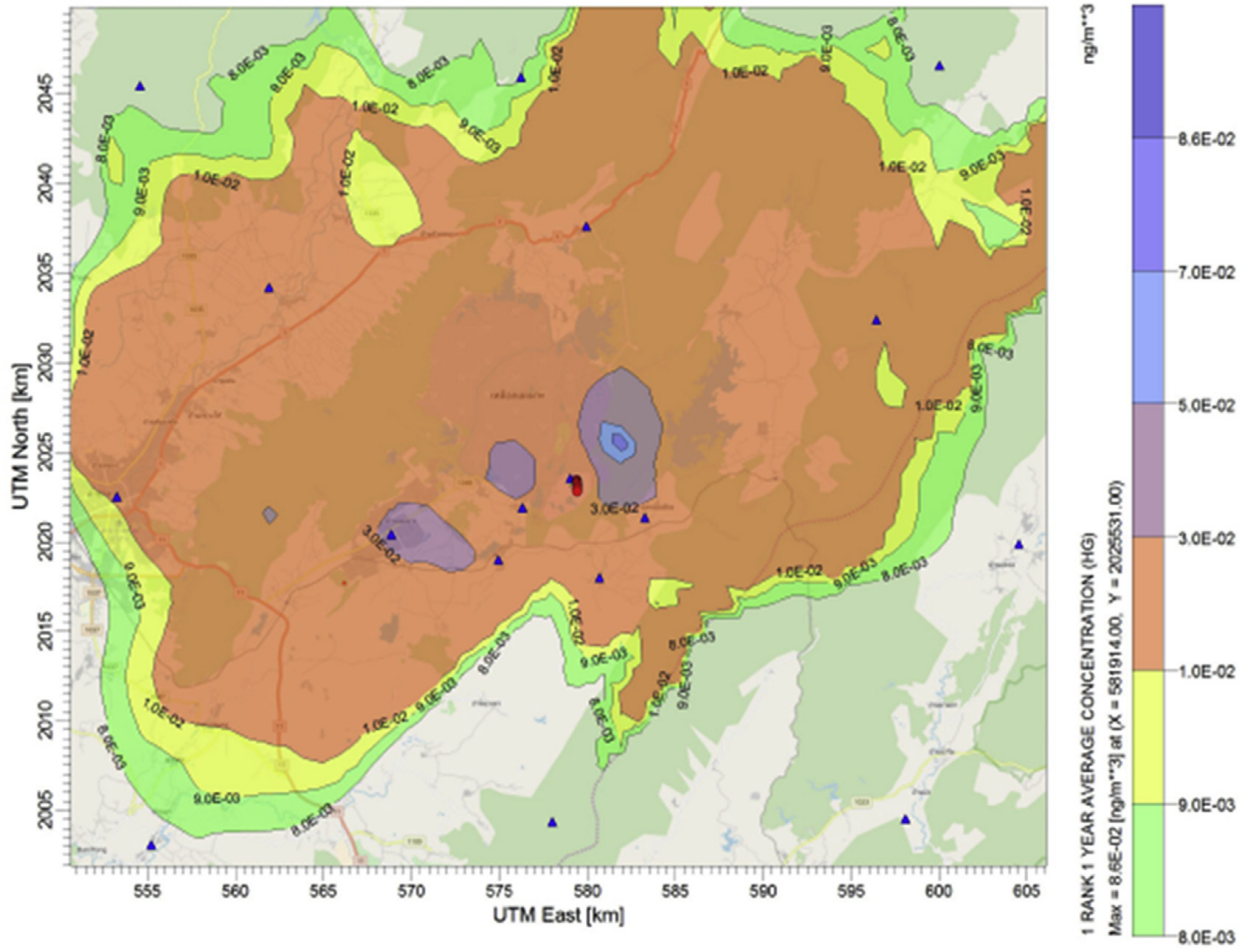
Annual average mercury concentration (ng/m^3^) under “ESPc + WFGD and coal washing scenario”.

**Figure 6:**
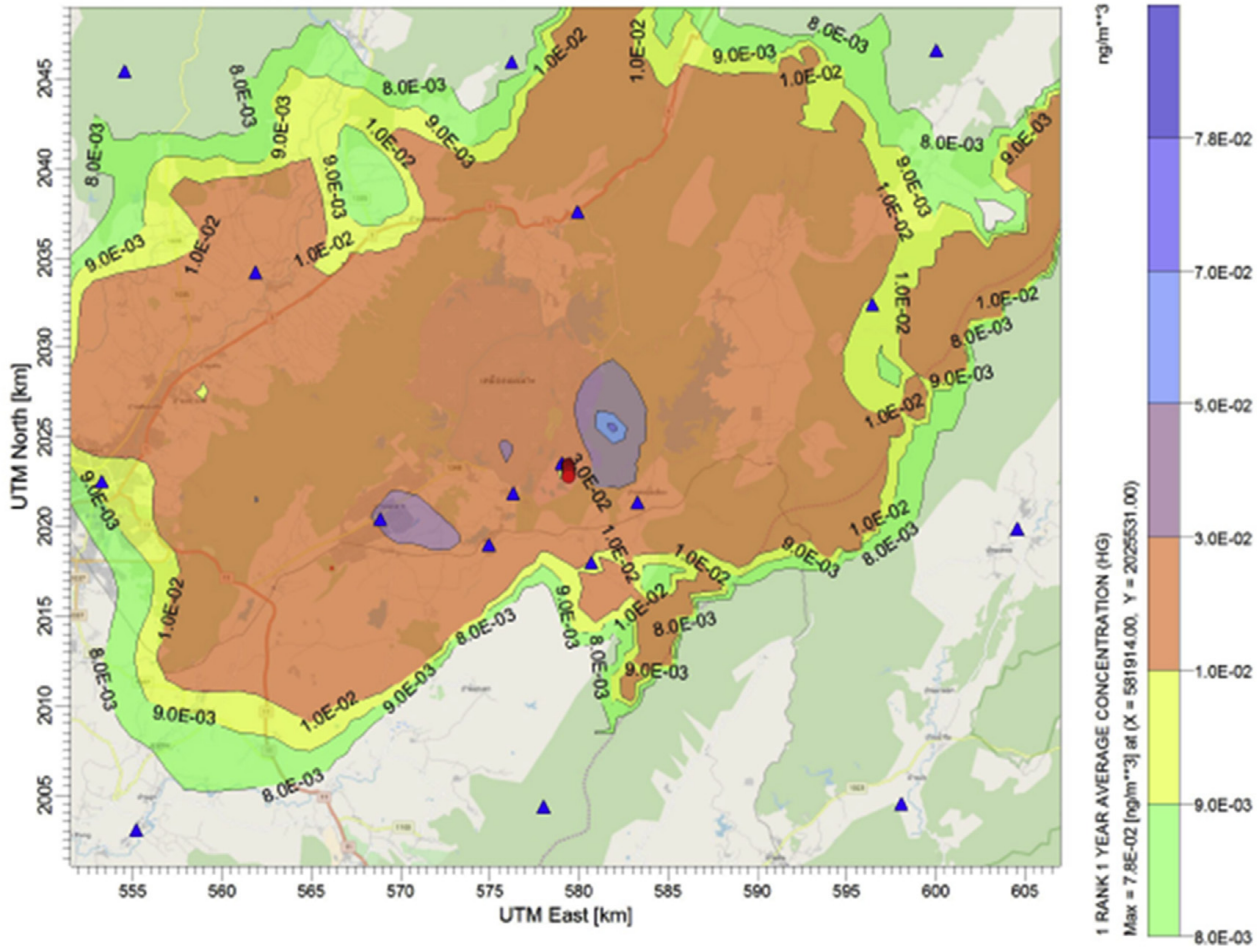
Annual average mercury concentration (ng/m^3^) under “ESPc + WFGD + Untreated ACl before ESPc scenario”.

Average ground level concentrations of mercury emitted from the power plant are predicted using the CALPUFF air dispersion model. Their annual levels simulated under each emission scenarios are compared with the current air emission control of the source (business as usual or BAU scenario). Results are illustrated in Figures 3, 4, 5 and 6. The maximum ground level concentration of the BAU scenario is predicted at 0.137 ng/m^3^. Predicted concentrations at the discrete receptor points are in the range of 0.00136 ng/m^3^ - 0.13673 ng/m^3^ as illustrated in Figure 3. However, without any air pollution control devices installed at the power plant, the maximum ground level concentration of mercury is predicted at 1.262 ng/m^3^ with the values of predicted concentrations at discrete receptors between 0.00817 ng/m^3^ - 1.2616 ng/m^3^ (Figure 4).

Adding of coal washing to the BAU can significantly reduce the emission hence the ambient concentration of mercury as presented in Figure 5. Average annual concentrations at the receptors are predicted in between 0.00085 ng/m^3^ - 0.0859 ng/m^3^. The maximum ground level concentration within the modeling domain is predicted at 0.086 ng/m^3^. This level is almost half of the concentration predicted from the BAU case since the coal washing can reduce the content of mercury in feed coal by at least 30% (Streets et al., 2005).

Untreated ACl installation is a post-combustion technology considered to have better performance in removing of mercury more than those pre-combustion and combustion control (Kilgroe et al., 2002; United Nations Environmental Programme, 2010). By adding the untreated ACI before ESPc in the BAU case, the maximum ground level concentration is predicted at 0.078 ng/m^3^ with the annual concentrations between 0.000781 ng/m^3^ to 0.07813 ng/m^3^ at the receptors (Figure 6).

Results from this study indicates that there is a strong relationship between changing of amount of mercury emission towards the decreasing of ambient mercury air concentrations. Decreasing of ambient concentrations were predicted to be achieved as a result from both the BAU scenario (installation of typical air pollution control devices) and from adding of specific mercury control system to the existing operation. However, in order to evaluate the level of optimum control of mercury emission, we further compare the predicted maximum ground level concentrations obtained from every scenarios with the available annual mercury concentration standard in order to evaluate the necessity of adding the mercury control to the existing air pollution control devices of the power plant. It is found that these levels are much lower than the Japanese's standard regulated at 40 ng/m^3^ (annual average). Therefore, it can be concluded from this study that it is unnecessary to have specifically additional mercury control measures to this power plant. This study also reveals that the typical air pollution control system currently installed at the power plant has a co-benefit in an appropriate level to reduce mercury emission. Results from this study demonstrate the need to evaluate such kind of co-benefit for effective management of the release of mercury and air pollution from the industry.

## 4 Conclusion

Coal-fired power plants are considered one of the major emission source of air pollutants. For the decades, this source is generally well managed in air pollution control by applying several emission control measures. This effort is mainly aimed to reduce the concentrations and emissions of conventional air pollutants. However, with the emerging concern on the health and ecological toxic related mercury, the coal-fired power plant acknowledged as one of the major emission source is also required to manage for the controlling of their mercury emission. This study is aimed to evaluate the appropriate controlling measures with can provide the effective benefit in managing of both emissions and ambient concentrations of mercury through the case study of the release from coal-fired power plant. The results clearly reveal the success of exiting conventional air pollution control devices which mainly used to control particulate matter, sulfur dioxide and oxide of nitrogen on their co-benefit in reducing the emission of mercury. Mercury emission is reduced from 374 g/h (without any air pollution control devices) to 41 g/h under this existing business as usual operation. We further evaluate for the need to add the specific mercury control system to the current operation by calculating the changing of emissions and ground level concentrations. Calculated results show deceasing of mercury emission at different levels according to various additional controlling measures. However, there are very small change of mercury predicted on the ground level ambient concentrations as compare with the exiting operation of the power plant. This study reveals the success of existing air pollution control devices currently installed at the power plant towards achievement of reducing of mercury emission in an appropriate level. Finding and methodology in this study can be used as a case study in quantitative evaluation of the effectiveness and appropriateness of environmental control mitigation measures added to the existing operations. It clearly illustrates the need to analyze the co-benefit of current air pollution control system towards the accomplishment on controlling emissions of other emerging air pollutants which will provide the best optimum air pollution control to the emission source.

## Declarations

### Author contribution statement

S.Thepanondh: Conceived and designed the experiments; Analyzed and interpreted the data; Wrote the paper.

V. Tunlathorntham: Performed the experiments; Contributed reagents, materials, analysis tools or data.

### Funding statement

This research did not receive any specific grant from funding agencies in the public, commercial, or not-for-profit sectors.

### Competing interest statement

The authors declare no conflict of interest.

### Additional information

No additional information is available for this paper.
